# Tim3 and PD-1 as a therapeutic and prognostic targets in colorectal cancer: Relationship with sidedness, clinicopathological parameters, and survival

**DOI:** 10.3389/fonc.2023.1069696

**Published:** 2023-03-23

**Authors:** Zahra Mokhtari, Marzieh Rezaei, Mohammad Hossein Sanei, Amirreza Dehghanian, Zahra Faghih, Zahra Heidari, Shirin Tavana

**Affiliations:** ^1^ Department of Immunology, School of Medicine, Isfahan University of Medical Sciences, Isfahan, Iran; ^2^ Department of Pathology, School of Medicine, Isfahan University of Medical Sciences, Isfahan, Iran; ^3^ Department of Pathology, School of Medicine, Shiraz University of Medical Science, Shiraz, Iran; ^4^ Institute for Cancer Research (ICR), School of Medicine, Shiraz University of Medical Sciences, Shiraz, Iran; ^5^ Department of Biostatistics & Epidemiologyt, School of Public Health, Isfahan University of Medical Sciences, Isfahan, Iran

**Keywords:** colorectal cancer, immune checkpoints, programmed cell death protein-1, T cell immunoglobulin and mucin-domain containing-3, immunotherapy, prognosis

## Abstract

**Background:**

Colorectal cancer (CRC) is a heterogeneous disease that complicates predicting patients’ prognosis and their response to treatment. CRC prognosis is influenced by the tumor microenvironment (TME). The immune system is a critical component of the TME. Programmed cell death receptor 1 (PD-1) and T-cell immunoglobulin and mucin-domain containing-3 (Tim3) are inhibitory immune checkpoints that regulate immune response and may provide prognostic power. However, the effect of their expressions and co-expressions on the CRC prognosis remains unclear. Accordingly, this study aimed to investigate the prognostic value of the CD8, CD3, PD-1, Tim3 expression, and PD-1/Tim3 co-expression in patients with CRC.

**Materials and Methods:**

One hundred and thirty six patients with CRC who underwent curative surgery were enrolled in the study. Immunohistochemical staining was performed for PD-1, Tim3, CD8, and CD3, and the expression of each marker was evaluated in the center of the tumor (CT), invasive margin (IM), and adjacent normal-like tissue.

**Result:**

Our results indicated that high expression of PD-1 in IM was significantly associated with lower TNM stage, T-stage, M-stage, lack of metastasis, the presence of tertiary lymphoid structure (TLS), lack of recurrence (in the left-sided tumors), and larger tumor size (in right-sided tumors) (P<0.05). High expression of PD-1 in IM was also associated with improved overall survival (OS) in a subgroup of patients with high CD8 expression. High Tim3 expression in CT was associated with higher M-stage (M1) (in left-sided CRCs) (P<0.05). It was also associated with decreased OS in total cohort and left-sided CRCs and represented an independent prognostic factor for CRC patients in multivariate analysis. PD-1 and Tim3 co-expression had no synergistic effects on predicting OS.

**Conclusion:**

Our findings suggest that the clinicopathological and prognostic significance of immune system-related markers such as CD8, PD-1, and Tim3 depends on the primary tumor sides. We also showed that Tim3 could act as a prognostic factor and therapeutic target in CRC. This marker is probably a more preferred target for immunotherapy than PD-1, especially in left-sided CRCs.

## Introduction

Colorectal cancer (CRC) is the third most common cancer and the second cause of cancer death globally (1). CRC is a complex, molecularly heterogeneous disease that is characterized by diverse genomic and immunologic landscapes. It shows different incidence, pathogenesis, molecular pathways, immunogenicity, and patient outcome depending on tumor location (2, 3). The inherent complexity of these multifactorial diseases dramatically complicates predicting patients’ prognoses and responses to treatment (4). AJCC/UICC-TNM is a standard method for the classification of malignant tumors. This method relies on tumor characteristics, including primary tumor extension (T), lymph node involvement (N), and distant metastasis (M)(6). Despite global acceptance, the importance and power of TNM-staging, it has some drawbacks as patients in the same stage, could have different clinical outcomes and prognosis (5, 6).

Nowadays, it is extensively accepted that the immune components of the tumor microenvironment (TME) play a critical role in tumor development (7). Accordingly, analysis of the interactive relationships between tumor cells and the immune system components in the TME have received more attention (8). Many studies have established that the high density of different T cell subpopulations such as CD3+ T cells, CD8+ T cells, and CD45RO+ memory T cells in tumor tissue is associated with more prolonged overall survival (OS) and disease-free survival (DFS) in different tumor types (8).

One of the critical regulatory molecules in the TME is immune checkpoints (ICPs) that regulate the functions of infiltrated immune cell. ICPs refer to both activatory and inhibitory molecules which act as gatekeepers of immune responses. The inhibitory checkpoints include, but are not limited to programmed cell death receptor 1 (PD-1), cytotoxic T-lymphocyte associated protein-4 (CTLA4), T cell immunoglobulin domain and mucin domain-containing-3 (Tim3), Lymphocyte Activating 3 (LAG3), T cell immunoreceptor with Ig and ITIM domains (TIGIT), and B- and T-lymphocyte attenuator (BTLA) and. Their expression in malignant tumors is often significantly increased and is associated with poor prognosis (9). But, little is understood about how comprehensive regulation patterns of ICP molecules relate to immune responses, TME formation, and patient outcomes (10).

PD-1 belongs to the immunoglobulin superfamily and is expressed on the activated and regulatory T cells (Treg), B cells, natural killer (NK) cells, NKT cells, macrophages, dendritic cells, and monocytes (11, 12). The engagement of PD-1 by its ligands, PD-L1 and PD-L2, induces tumor immune escape and down-regulation of tumor-infiltrating lymphocytes (TILs) by different mechanisms such as: inhibiting T cell proliferation and induction of their apoptosis, reducing the inflammatory cytokines as IFN-γ, IL-2, TNF-α, inhibiting granular enzyme and perforin production by cytotoxic T lymphocytes (CTLs), and increasing metastasis and penetration of tumor cells (12–14). Immune checkpoint inhibitors (ICIs) targeting PD-1 or PD-L1 have shown objective responses in certain cancers, including melanoma, renal cell carcinoma, and non-small cell lung cancer (15). Pembrolizumab and nivolumab, as PD-1-blocking antibodies, have also been accelerated FDA approval after showing their effectiveness in the patients with metastatic CRC (16). While PD-1 showed inhibitory and therapeutic effects, there are pieces of conflicting evidence on its prognostic significance in CRC (17–23).

Tim3is another immunoglobulin superfamily co-inhibitory receptor expressed on both immune and tumor cells, including CTLs, type 1 T helper (Th1) cells, Th17, Tregs, and innate immune cells (24). Described ligands for Tim3 are high-mobility group protein B1 (HMGB1), galectin 9, carcinoembryonic antigen cell adhesion molecule 1 (CEACAM1), and phosphatidylserine (PtdSer) (25). Even though the Tim3 intracellular signaling pathways have not been fully elucidated, it appears that they finally lead to inhibiting antitumor immune responses by blocking effector T cell responses, increasing Treg functions, and growing myeloid-derived suppressor cells (MDSCs) inside tumors (26–28). A growing number of studies have shown that Tim3 up-regulation in tumor tissue is associated with poor prognosis in a wide variety of cancers such as CRC, gastric cancer, prostate cancer, clear cell renal cancer, urothelial bladder cancer, and cervical cancer (29–34). Thus, Tim3 could be introduced as a potential prognostic factor and a new target for immunotherapy in solid tumors (35). In CRC, Tim3 expression has been reported to be higher in tumor tissues than in normal tissues and is significantly associated with advanced stages and metastasis (36). However, the prognostic value of Tim3 expression in tumor tissue or immune cells in patients with CRC isn’t well documented (20, 29, 37). Previous studies have shown that though Tim3 expression is associated with T cell exhaustion in cancer patients, the co-expression of Tim3 with PD-1 represents more exhausted CD4+ and CD8+ T cells (38, 39). Consistently, preclinical studies demonstrated that co-blockade of Tim3 and PD-1 pathways is highly effective in the treatment of solid tumors (40).

Accordingly, we hypothesized that the PD-1 and Tim3 expression could be associated with poor prognosis and their co-expression would be a robust prognostic marker that besides the TNM staging system can be useful in dividing patients into more homogeneous groups. Moreover, according to the different characteristics of the right and left-sided CRCs, the prognostic impact of PD-1 and Tim3 would be different on each tumor side. Therefore, in this study, we also examined the expression of these markers in the center (CT) and invasive margin (IM) of tumor tissues in a total cohort and subdivided left and right-sided CRCs to determine whether they have any relations with clinicopathological parameters and prognosis.

## Materials and methods

### Patients

A total of 136 patients with CRC, operated between 2013 and 2016 at the Alzahra Hospital (Isfahan, Islamic Republic of Iran), were selected and retrospectively analyzed. Patients with preoperative chemotherapy, having a history of other cancers or autoimmune diseases, insufficient, and inappropriate tissue were excluded. Hematoxylin and eosin (H&E) slides were reviewed by an expert pathologist to confirm the patient’s pathological data and to select the best tissue block that simultaneously contained the center and invasive margin of the tumor. Pathological data including TNM stage (according to AJCC, 8th edition), lymph node metastasis, tumor differentiation, lymphovascular or perineural invasion, tumor budding, and tertiary lymphoid structure (TLS), as well as survival status, recurrence status, recurrence date, and for patients who had died, date and cause of death, were obtained from H&E slides, their electronic medical records, and direct phone contact with their relatives.

### Immunohistochemistry

Immunohistochemical (IHC) staining was performed on 4-μm sections of paraffin-embedded tissues. Paraffinized sections were baked overnight at 45°C, de-paraffinized in xylene (Merk, Germany), rehydrated in graded ethanol (Merk), processed for antigen retrieval by high-pressure cooking in Tris-EDTA antigen retrieval solution (pH=9) for 25 min, and quenched for endogenous peroxidase activity in peroxidase blocking reagent (Master Diagnostica, Spain) at room temperature (RT) for 10 min. Then, the sections were incubated with anti-human antibodies against PD-1 (1:150, SB-019261, Sina Biotech, Iran) at RT for 1 hour, and Tim3 (1:2000, ab241332, Abcam, USA), CD3 (ready to use, 1:2.5, MAD-000621QD, Master diagnostica), CD8 (1:150, 372902, Biolegend, USA) overnight at 4°C in a moisture chamber. Immunostaining was performed using Master Polymer Plus Detection System (Master Diagnostica) resulted in a brown-colored precipitate at the antigen site. To do this, following incubation time, the sections were treated with a Primary Antibodies Amplifier Master for 15 min, followed by Master Polymer Plus HRP for 40 min at RT. Diaminobenzidine (DAB) solution was added as substrate. Finally, the sections were subjected to hematoxylin staining, dehydration, and mounting with Entellan (Merck). Tonsil sections were used as a positive control for all antibodies. The primary antibodies omitted were used as negative controls.

### Evaluation

Two experienced pathologists who were blinded to the clinical results of patients evaluated the stained slides. First, all slides belonging to each patient were examined with ×100 magnification, and normal-like areas, invasive margin (IM), and center of the tumor (CT) were identified. Then, each area was checked with ×100 magnification, and an area representing the average expression was selected. Next, the selected area was further reviewed with ×400 magnification, and the percentage of cells expressing each marker was separately determined. The cells with bothmembranous or cytoplasmic staining for the markers were counted as positive. For PD-1, CD8, and CD3 markers, the percentage considered the ratio of positive lymphocytes to the total TILs in each region, but for Tim3, it represented the ratio of positive immune cells (adaptive and innate) to stromal cells. Since PD-1 and Tim3 expression had significant differences in the intensity among different samples, the intensity for each marker was also reported as low, intermediate, and high.

### Statistical analysis

Statistical analysis was conducted using SPSS software version 24 (IBM SPSS, USA). Chi-squared or Fisher’s exact tests were used to analyze categorical variables. To evaluate the association between PD-1 or Tim3 expression and clinicopathological characteristics, first, the normality of data was checked and abnormal distributed data were normalized by Ln or Sqrt, then an independent sample T-test was used. Paired t-test was used to compare the mean expression of Tim3 and PD-1 between CT, IM, and normal-like regions. Patients were also divided into high and low expression groups for CD8 (IM median= 25%, CT median= 15%) and CD3 (IM= 45%, CT= 40%) expression according to their median expression. For PD-1 and Tim3, the percentage and intensity of expression were also combined, and the patients were grouped into high and low groups using R software version 4.0.3. P-values of <0.05 were considered statistically significant. OS was defined as the time interval between surgery and death for any reason or last follow-up. DFS was measured as the time interval between surgery and CRC recurrence or metastasis. Both univariate and multivariate survival analysis were performed by Cox regression proportional hazard models to determine prognostic factors predicting OS and DFS. The multivariable analysis was conducted only on independent variables with a P-value of <0.10 in univariable analysis.

## Result

### Patient characteristics and clinical outcomes

One hundred and thirty six patients with CRC and a mean age ( ± SD) of 62.35 ± 14.10 years (range 19-92) at the time of diagnosis were included in this study. Patients were divided into two main categories based on their tumor location: right-sided (caecum, ascending, hepatic flexure, transverse colon) and left-sided (splenic flexure, descending, sigmoid colon, rectosigmoid, and rectum). Most patients were in pathological stages II (n= 46, 33.8%), and stage III (n= 40, 29.4%). 124 cases (91.2%) had no distant metastasis (M0) at the time of surgery. The complete patient’s demographic characteristics are summarized in **Table 1**.

**Table 1:** Demographics characteristics of the patients.

Parameters	No. of Cases (%)	Mean ± SD	Parameters	No. of Cases (%)
Total	136		TNM stage	
**Sex**			I/II	84 (61.8)
Male	83 (61.0)		III/IV	52 (38.2)
Female	53 (39.0)		**Lymphovascular invasion (LVI)**	
**Age**		62.35 ± 14.102	Absent	79 (58.1)
<63	65 (47.8)		Present	57 (41.9)
≥63	71 (52.2)		**Perineural invasion**	
**Tumor side**			Absent	111 (81.6)
Right	56 (41.2)		Present	25 (18.4)
Left	76(55.9)		**Metastasis**	
Unknown	4 (2.9)		Absent	89 (65.4)
**Tumor size**		5.56 ± 2.47	Present	34 (25.0)
<5	56 (41.2)		Unknown	13 (9.6)
≥5	78 (57.4)		**Recurrence**	
Unknown	2 (1.5)		Absent	97(71.3)
**Differentiation grade**			Present	29(21.3)
Low grade	76 (55.9)		Unknown	10(7.4)
Moderate to high grade	60 (44.1)		Tumor budding	
**T stage**			Low	89 (65.4)
T1/T2	47 (34.6)		High	47 (34.6)
T3/T4	89 (65.4)		**Tertiary lymphoid structure (TLS)**	
**Lymph node involvement**			Absent	104 (76.5)
Absent	90 (66.2)		Present	32 (23.5)
Present	46 (33.8)		**Survival**	
**M stage**			Alive	79 (58.1)
M0	124 (91.2)		Dead	57 (41.9)
M1	12 (8.8)			

### Distribution of cases according to high expression of PD1, Tim3, CD8, CD3 in CRC tumor tissue

**Figure 1:**
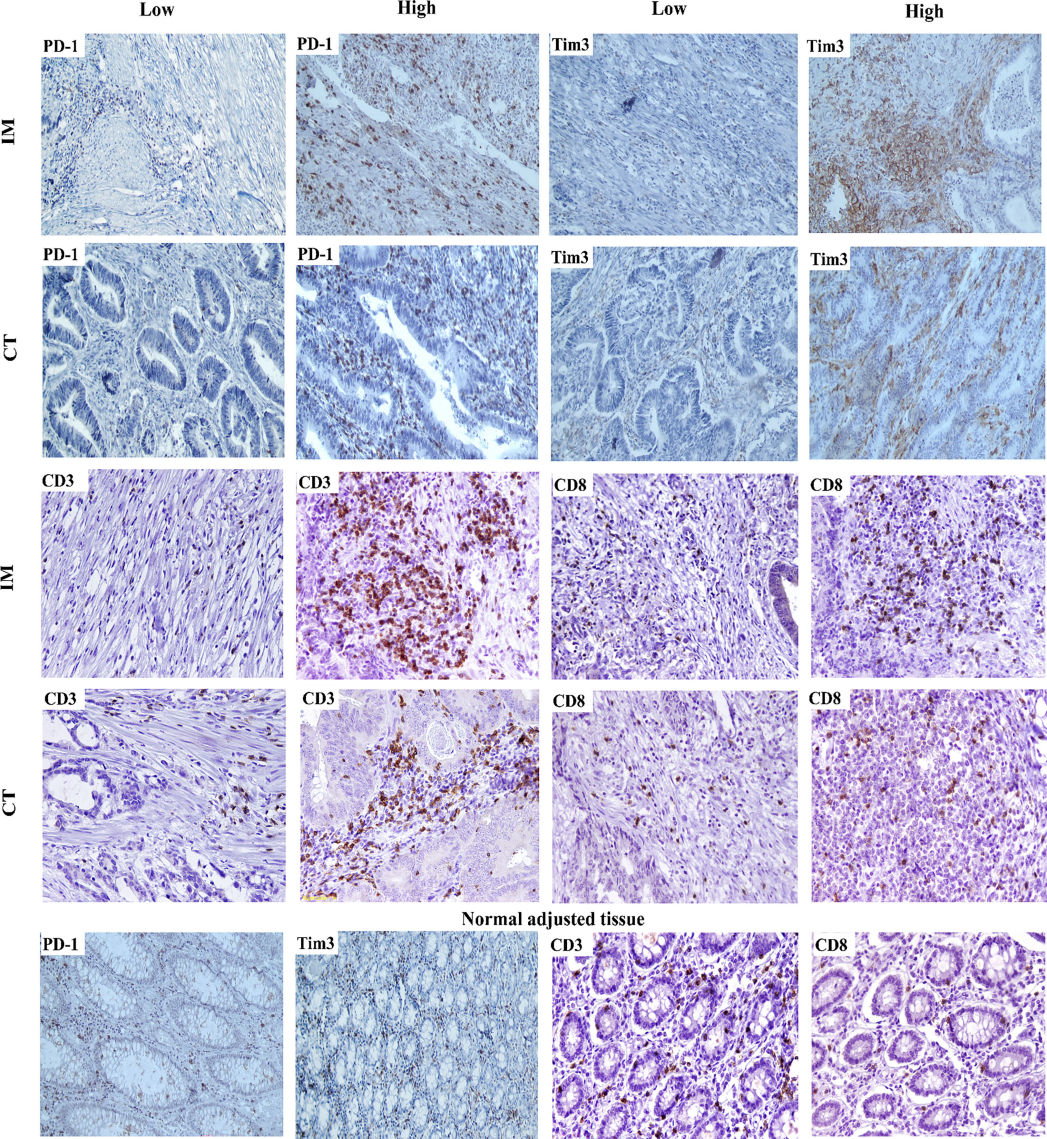
IHC staining of PD-1, Tim-3, CD3, and CD8 expressions in CRC (200×).

We first determined the percentage of infiltration of CD3+, CD8+, or PD-1+ TILs and Tim3+ TIICs within the CT, IM, and normal-like adjacent tissue using IHC methods. Our analysis revealed that all of the patients with CRC expressed PD-1 and Tim3 in their tumor tissues and the normal-like adjacent with various degree from high to low (**Figure 1**). Expressions of investigated markers in different regions (CT, IM, normal-like adjacent tissue) with details in total cases and based on the primary tumor side are shown in **Tables 2**, **3**, respectively.

**Table 2:** Distribution of cases according to the expression of PD-1, Tim3, CD3, and CD8 markers.

IHC Markers	Total cohort								
	Total	CT		Total	IM		Total	Normal-like adjacent tissue	
		Low (%)	High (%)		Low (%)	High (%)		Low (%)	High (%)
PD-1	136	83 (61)	53 (39)	135	63 (46.7)	72 (53.3)	61	36 (59.0)	25 (41.0)
Tim3	136	78 (57.4)	58 (42.6)	135	37 (27.4)	98 (72.6)	61	38 (62.3)	23 (37.7)
CD3	136	81 (59.6)	55 (40.4)	136	75 (55.1)	61 (44.9)	52	28 (53.8)	24 (46.2)
CD8	135	69 (51.1)	66 (48.9)	136	85 (62.5)	51 (37.5)	50	39 (78.0)	11 (22.0)

CT, Center of the tumor.

IM, Invasive margin of the tumor.

**Table 3:** Distribution of CD3+, CD8+, and PD-1+ TIL and Tim3+ TIICs at IM and CT according to the primary tumor side.

IHC Markers	Right-side						Left-side					
	Total	CT		Total	IM		Total	CT		Total	IM	
		Low (%)	High (%)		Low (%)	High (%)		Low (%)	High (%)		Low (%)	High (%)
PD-1	56	36(64.3)	20(35.7)	55	20(36.4)	35(63.6)	76	45(59.2)	31(40.8)	76	40(52.6)	36(47.4)
Tim3	56	32(57.1)	24(42.9)	56	11(19.6)	45(80.4)	76	42(55.3)	34(44.7)	75	25(33.3)	50(66.7)
CD3	56	35(62.5)	21(37.5)	56	29(51.8)	27(48.2)	76	43(56.6)	33(43.4)	76	45(59.2)	31(40.8)
CD8	56	31(55.4)	25(44.6)	56	36(64.3)	20(35.7)	75	35(46.7)	40(53.3)	76	46(60.5)	30(39.5)

CT, Center of the tumor.

IM, Invasive margin of the tumor.

### Mean expression of PD-1 and Tim3 in the center and invasive margin of the tumor and normal-like tissue

A comparison of patients that had both CT and IM (n= 135) indicated that the mean expressions of PD-1 and Tim3 in CT were significantly lower than in IM (P= 0.009 and P< 0.001, respectively). The expression of these markers in normal-like adjacent tissues (n= 61) also showed that the mean expression of PD-1 and Tim3 in both tumor regions were significantly higher than in normal-like tissue (P< 0.001) (**Table 4**).

**Table 4:** The mean expression of PD-1 and Tim3 in the center of the tumor, invasive margin, and normal-like tissue and their comparison.

		n	Mean ± SD	P
Pair 1	PD-1.CT	135	8.4519 ± 8.62893	0.009*^a^
	PD-1.IM	135	9.1481 ± 6.96636	
Pair 2	PD-1.CT	61	9.1967 ± 9.85024	<0.001*^a^
	PD-1.Normal	61	2.7049 ± 2.44502	
Pair 3	PD-1.IM	61	9.3279 ± 6.39980	<0.001*^a^
	PD-1.Normal	61	2.7049 ± 2.44502	
Pair 4	Tim3.CT	135	29.0815 ± 15.71721	<0.001*
	Tim3.IM	135	35.3407 ± 17.11252	
Pair 5	Tim3.CT	61	26.8852 ± 13.78900	<0.001*
	Tim3.Normal	61	14.4754 ± 10.95994	
Pair 6	Tim3.IM	60	34.9167 ± 17.74326	<0.001*
	Tim3.Normal	60	14.7167 ± 10.88786	

CT, Center of the tumor.

IM, Invasive margin of the tumor.

*Statistically significant.

a A T-test performed with normalized data (Ln or Sqrt) to obtain the P value.

### Expression of PD-1 and Tim3 in patients with different clinicopathological parameters

We then compared the mean expression of PD-1 and Tim3 in patients with different clinicopathological features. Our statistical analysis revealed that the mean expression of PD-1 in the CT was significantly higher in the patients with lower T stage (T1/T2; P= 0.047) and no metastasis (P=0.035). The mean expression of PD-1 in IM was also significantly higher in patients with larger tumor size (≥ 5cm) (P=0.027), M0-stage (P=0.044), no metastasis after surgery (P<0.001), no recurrence (P= 0.043), and patients with TLS (P= 0.002) (**Supplementary Table 1**).

Regarding Tim3 expression, the mean expression of Tim3 in the CT was significantly higher in females than males (P= 0.024). The mean expression of Tim3 in IM was also higher in right-sided CRCs (P= 0.032), patients with no metastasis (P= 0.038), and with TLS (P= 0.039) compared with left-sided CRCs, patients with metastasis and without TLS (**Supplementary Table 1**).

Patients were also divided into high and low expression groups for PD-1 and Tim3, based on their percentages and intensities, and the analysis were repeated. Our analysis indicated that high PD-1 expression in IM was associated with lower T-stage (T1/2; P= 0.032), lower M-stage (M0; P= 0.039), lower TNM-stage (I/II; P= 0.042), lack of metastasis (P= 0.001), and the presence of TLS (P= 0.045). No association was found between PD-1 expression in CT and clinicopathological parameters. While high Tim3 expression in CT was observed in elder patients (≥63; P= 0.047) and dead patients (P= 0.019). Tim3 expression in the IM was also higher in females (P= 0.029) and those patients with TLS (P= 0.03) (**Supplementary Tables 2, 3**).

### Association of PD1 and Tim3 expression with clinicopathological parameters based on the primary tumor side

We then classified patients based on their primary tumor side (left and right) and assessed the association of PD-1 and Tim3 expression with different clinicopathological parameters (**Supplementary Tables 2, 3**). Similar to total cohort, high PD-1 expression in IM was associated with lower T-stage (T1/2) in left-sided CRCs (P= 0.023), lower M-stage (M0) in right-sided CRCs (P= 0.014), lack of metastasis in both sides (P= 0.047 for right-sided and P= 0.01 for left-sided), larger tumor size (≥ 5 cm) in right-sided CRCs (P= 0.027), lack of recurrence in left-sided CRCs (P= 0.03). High Tim3 expression in CT was associated with older age in left-sided CRCs (P= 0.006), survival status (dead) in left-sided CRCs (P= 0.024) and with higher M-stage (M1) in left-sided CRCs (P= 0.019). High Tim3 expression in the IM was associated with the presence of TLS in right-side CRCs (P= 0.049).

### Correlation of PD-1 and Tim3 expression with each other and with lymphocytes density

As summarized in **Supplementary Tables 2, 3**, in the total cohort, there were significant direct relationships between PD-1 and Tim3 expression and the presence of CD3+ or CD8+ cells in the different tumor regions. Just the correlation between Tim3 and CD8 expression was not significant. These results were also obtained in both left/right-sided tumors, separately.

### Survival analysis

**Figure 2:**
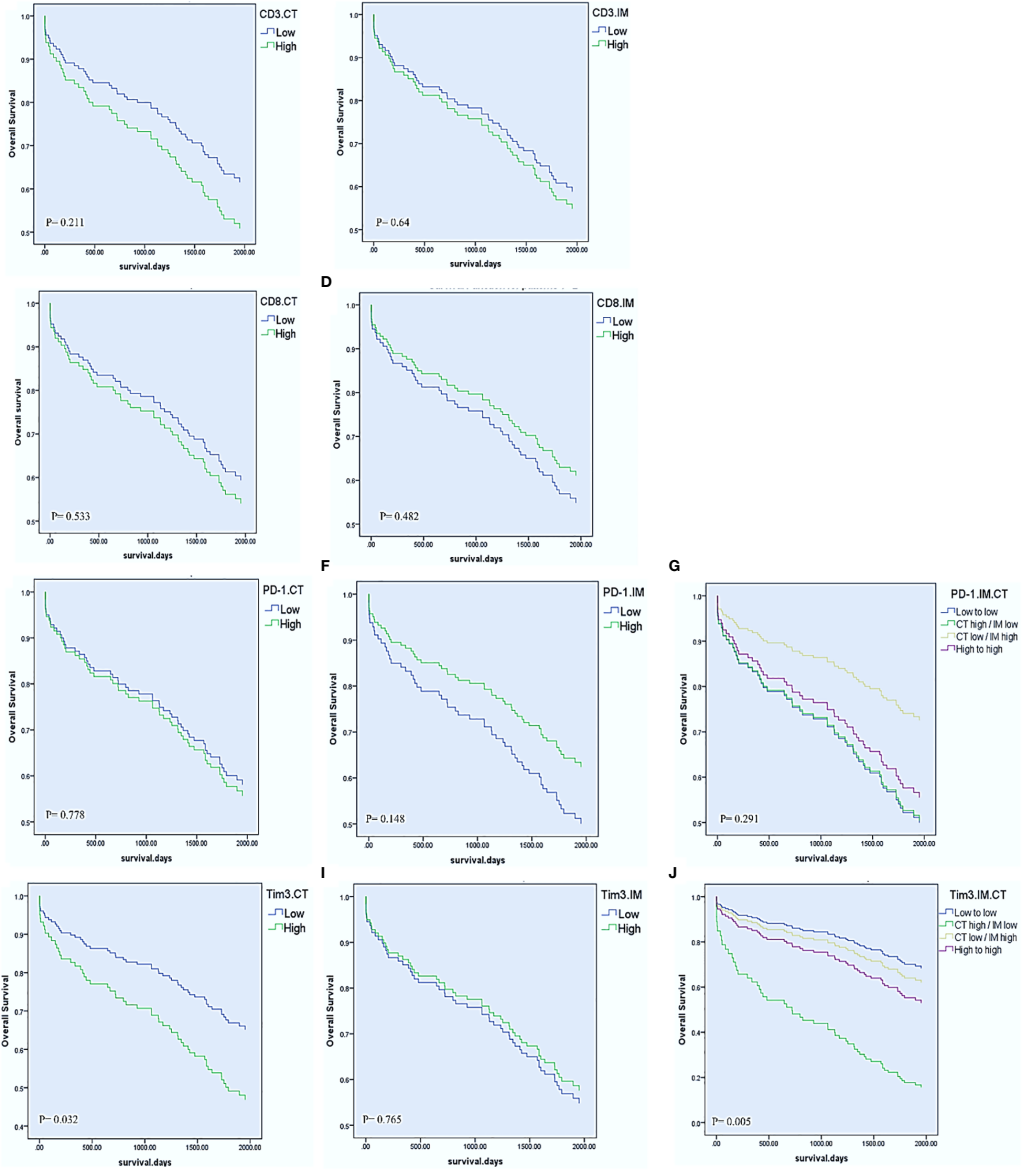
Univariable Cox regression estimates of overall survival according to CD8, CD3, PD-1, and Tim3 high vs low expression in the center of the tumor (CT) and invasive margin (IM). CD3 and CD8 in the CT and IM **(A–D)**. PD-1 expression in the CT, IM, and combination of IM with CT **(E–G)**. Tim3 expression in the CT, IM, and combination of IM with CT **(H–J)**.

The mean time of follow-up (± SD) was 1622.6 ± 830.45 days. In the last follow-up, 79 patients (58.1%) were alive, and 97 patients (71.3%) had no recurrence experience. Cox regression proportional hazard models were performed to, beside clinicopathological parameters, evaluate the effects of PD-1, Tim3, CD3, and CD8 expression in CT and IM on OS and DFS in the patients with CRC. For the total cohort, the univariable Cox regression model revealed that Tim3 expression in CT (HR= 1.769, 95% CI= 1.050-2.980, P= 0.032), lymph node involvement (HR= 2.713, 95% CI= 1.611-4.568, P< 0.001), M-stage (HR= 7.443, 95% CI= 3.741-14.808, P< 0.001), and TNM-stage (HR= 3.529, 95% CI= 2.074-6.006, P< 0.001) were significantly associated with shorter OS (**Table 5**, **Figure 2**). The univariate Cox regression analysis for DFS revealed no significant relationship between the expression of any of investigated markers and the risk of post-operative disease relapse (data not shown).

**Table 5:** Univariable survival analysis in the total cohort.

Parameters	Univariable analysis HR (95% CI)	P	Parameters	Univariable analysis HR (95% CI)	P
**Sex**			**Tertiary lymphoid structure (TLS)**		
Male	1	0.174	Absent	1	0.415
Female	0.679 (0.388-1.187)		Present	0.760 (0.394-1.469)	
**Age**			**PD-1.CT**		
<63	1	0.799	Low	1	0.778
≥63	1.070 (0.635-1.802)		High	1.079 (0.636-1.832)	
**Tumor side**			**PD-1.IM**		
Right	1	0.119	Low	1	0.148
center	1.569 (0.891-2.762)		High	0.681 (0.405-1.146)	
**Tumor size**			**Tim3.CT**		
<5	1	0.549	Low	1	0.032*
≥5	1.178 (0.689-2.015)		High	1.769 (1.050-2.980)	
**Differentiation grade**			**Tim3.IM**		
Low grade	1	0.205	Low	1	0.765
Moderate to high grade	1.399 (0.832-2.353)		High	0.917 (0.520-1.618)	
**T stage**			**CD3.CT**		
T1/T2	1	0.06	Low	1	0.211
T3/T4	1.760 (0.976-3.176)		High	1.394 (0.828-2.344)	
**Lymph node involvement**			**CD3.IM**		
Absent	1	<0.001*	Low	1	0.64
Present	2.713 (1.611-4.568)		High	1.133 (0.672-1.908)	
**M stage**			**CD8.CT**		
M0	1	<0.001*	Low	1	0.533
M1	7.443 (3.741-14.808)		High	1.180 (0.702-1.983)	
**TNM stage**			**CD8.IM**		
I/II	1	<0.001*	Low	1	0.482
III/IV	3.529 (2.074-6.006)		High	0.821 (0.473-1.424)	
**Lymphovascular invasion (LVI**)			**PD-1.IM/CT**		
Absent	1	0.663	Low to low	1	0.291
Present	1.123 (0.666-1.896)		CT high/IM low	0.986 (0.344-2.827)	0.98
**Perineural invasion**			CT low/IM high	0.462 (0.209-1.021)	0.056
Absent	1	0.14	High to high	0.848 (0.469-1.532)	0.584
Present	1.576 (0.862-2.881)		**Tim3.IM/CT**		
**Tumor budding**			Low to low	1	0.005
Low	1	0.065	CT high/IM low	4.884 (1.874-12.733)	0.001
High	1.643 (0.970-2.783)		CT low/IM high	1.256 (0.560-2.817)	0.581
			High to high	1.669 (0.772-3.608)	0.193

CT, Center of the tumor.

IM, Invasive margin of the tumor.

*Statistically significant.

In the next step, we entered independent variables with P-value less than 0.1 of the univariate analysis to a multiple Cox regression model. Two different models were evaluated. In the first model: T-stage, lymph node involvement, M-stage, tumor budding and Tim3 expression in CT were entered and showed that lymph node involvement (HR= 2.705, 95% CI= 1.576-4.641, P< 0.001), M-stage (HR= 5.949, 95% CI= 2.863-12.361, P< 0.001), and Tim3 expression in CT (HR= 1.732, 95% CI= 1.005-2.985, P= 0.048) were independent prognostic factors for OS. In the second model: TNM-stage, tumor budding, and Tim3 expression in CT were entered and showed that TNM-stage (HR= 3.504, 95% CI= 2.030-6.047, P< 0.001) and Tim3 expression in CT (HR= 1.799, 95% CI= 1.057-3.065, P= 0.031) were remained in the equation as independent prognostic factors for shorten OS (**Table 6**).

**Table 6:** Multivariable Cox regression survival analysis.

Models	Parameters	Multivariable analysis	P
		HR (95% CI)	
**Model.1**	**M stage**		
	M0	1	<0.001*
	M1	5.949 (2.863-12.361)	
	**T stage**		
	T1/2	1	0.661
	T3/4	1.154 (0.608-2.193)	
	**Lymph node involvement**		
	Absent	1	<0.001*
	Present	2.705 (1.576-4.641)	
	**Tumor budding**		
	Low	1	0.748
	High	1.097 (0.624-1.929)	
	**Tim3.CT**		
	Low	1	0.048*
	High	1.732 (1.005-2.985)	
**Model.2**	**TNM stage**		
	I/II	1	<0.001*
	III/IV	3.504 (2.030-6.047)	
	**Tumor budding**		
	Low	1	0.591
	High	1.162 (0.672-2.012)	
	**Tim3.CT**		
	Low	1	0.031*
	High	1.799 (1.057-3.065)	

CT, Center of the tumor.

IM, Invasive margin of the tumor.

*Statistically significant.

When the patients were stratified by tumor side, we observed that high CD8 expression in IM in right-sided CRCs was significantly associated with favorable OS (HR= 0.288, 95% CI= 0.083-0.995, P= 0.049) while Tim3 in the left-sided CRCs was significantly associated with poorer OS (HR= 2.064, 95% CI= 1.063-4.007, P= 0.032) (**Table 7**).

**Table 7:** Cox regression survival analysis according to right and left-sided subgroups.

Parameters	Total cohort		Right-side		Left-side	
	Univariable analysis HR (95% CI)	P	Univariable analysis HR (95% CI)	P	Univariable analysis HR (95% CI)	P
PD-1.CT						
Low	1	0.778	1	0.851	1	0.998
High	1.079 (0.636-1.832)		0.91 (0.342-2.426)		1.001 (0.516-1.942)	
PD-1.IM						
Low	1	0.148	1	0.292	1	0.463
High	0.681 (0.405-1.146)		0.606 (0.239-1.537)		0.781 (0.405-1.510)	
Tim3.CT						
Low	1	0.032*	1	0.23	1	0.032*
High	1.769 (1.050-2.980)		1.768 (0.697-4.483)		2.064 (1.063-4.007)	
Tim3.IM						
Low	1	0.765	1	0.682	1	0.449
High	0.917 (0.520-1.618)		1.296 (0.375-4.478)		0.772 (0.395-1.509)	
CD3.CT						
Low	1	0.211	1	0.521	1	0.359
High	1.394 (0.828-2.344)		1.356 (0.535-3.441)		1.358 (0.706-2.610)	
CD3.IM						
Low	1	0.640	1	0.409	1	0.444
High	1.133 (0.672-1.908)		0.671 (0.26-1.73)		1.293 (0.670-2.496)	
CD8.CT						
Low	1	0.533	1	0.938	1	0.578
High	1.180 (0.702-1.983)		1.037 (0.409-2.629)		1.205 (0.624-2.327)	
CD8.IM						
Low	1	0.482	1	0.049*	1	0.499
High	0.821 (0.473-1.424)		0.288 (0.083-0.995)		1.257 (0.648-2.440)	

CT, Center of the tumor.

IM, Invasive margin of the tumor.

*Statistically significant.

We also combined the expression levels of PD-1 or Tim3 in IM and CT to further compare the effects of their expression on OS. Accordingly, we obtained four groups: 1. low expression in CT and IM, 2. high expression in CT but low expression in IM, 3. low expression in CT but high expression in IM, 4. high expression in both CT and IM. The results showed that those patients with high expression of Tim3 in CT and low expression in IM had significantly poorer OS than other patients (P= 0.005). On the other hand, patients with low expression of PD-1 in CT and high expression in IM had better OS than other patients; however it was not statistically significant (P= 0.291) (**Table 5**, **Figure 2**).

When patients were sub-classified based on CD8 expression, high PD-1 expression in IM showed significant association with prolonged OS in patients with high CD8 expression in this region (HR= 0.343, 95% CI= 0.138-0.852, P= 0.021). In addition, high Tim3 expression in CT was significantly associated with poor OS in patients with high CD8 expression in this region (HR= 3.032, 95% CI= 1.338-6.869, P= 0.008) (detailed in **Table 8**).

**Table 8:** Cox regression survival analysis according to CD8 high and low subgroups.

Parameters	CD8.CT-Low		CD8.CT-High			CD8.IM-Low		CD8.IM-High	
	Univariate analysis HR (95% CI)	P	Univariate analysis HR (95% CI)	P	Parameters	Univariate analysis HR (95% CI)	P	Univariate analysis HR (95% CI)	P
**PD-1.CT**					**PD-1.IM**				
Low	1	0.937	1	0.841	Low	1	0.967	1	0.021*
High	0.968 (0.426-2.198)		1.077 (0.520-2.232)		High	0.987 (0.520-1.870)		0.343 (0.138-0.852)	
**Tim3.CT**					**Tim3.IM**				
Low	1	0.956	1	0.008*	Low	1	0.707	1	0.727
High	0.978 (0.451-2.121)		3.032 (1.338-6.869)		High	0.881 (0.456-1.704)		1.246 (0.363-4.280)	

CT, Center of the tumor.

IM, Invasive margin of the tumor.

*Statistically significant.

**Figure 3:**
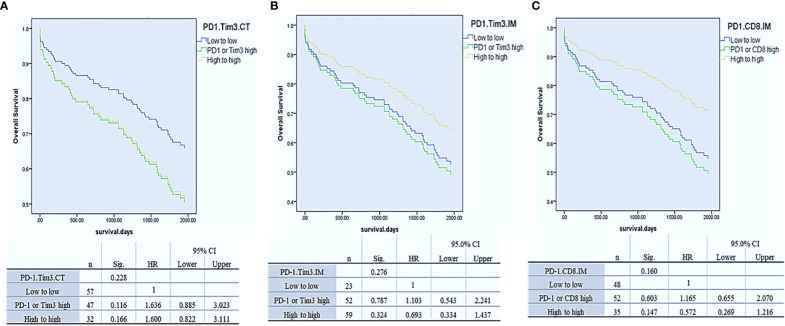
Cox-regression survival analysis of PD-1/Tim3 co-expression in the center of the tumor (CT, **A**) and invasive margin of the tumor (CT, **B**) and analysis of PD-1/CD8 co-expression in the IM **(C)**.

To assess whether Tim3 and PD-1 expressions had a synergistic effect in predicting OS, as previous studies showed (20, 37), we divided patients into three groups separately in each region: 1. Low expression of PD-1 and Tim3, 2. high expression of PD-1 or Tim3, and 3. high expression of PD-1 and Tim3. Our results demonstrated that the expression of these two markers had a synergistic effect in predicting OS, though it was not significant (P= 0.228 for CT, HR, CI, P= 0.276 for IM) The best OS was observed in patients with high PD-1 and Tim3 expression in IM but low PD-1 and Tim3 expression in the CT implying that their synergistic effect depends on the tumor region (more details in **Figure 3**).

Since the expression of PD-1 and CD8 in IM displayed a similar effect on OS, we hypothesized that their co-expression would synergistically predict survival, as a previously reported (19). Thus, we combined their expression in this region, and three groups of patients were compared: PD-1 and CD8 low, PD-1 or CD8 high, and PD-1 and CD8 high. Analysis revealed that the expression of these two markers had a synergistic effect in predicting OS, as well. Patients with high PD-1 and CD8 expression in IM had better OS than others though it was not statistically significant (P= 0.16) (**Figure 3**).

## Discussion

The fundamental, undeniable role of the immune system in tumor evolution and progression has prompted the evaluation of immune cell infiltration, as a prognostic marker for solid tumors, including CRC (41). Studies have shown that high infiltrations of CD3+ and CD8+ T cells in the TME are associated with improved DFS and OS in patients with CRC (8). Accordingly, in the present study, we assessed the CD3 and CD8 expressions in the IM and CT to evaluate their prognostic potential in CRC, however similar to Barbosa et al. (42), no statistically significant association was found in the total cohort. Since proximal and distal CRC displays distinct epidemiological, pathological, and clinical entities (2, 3), we further analyzed TILs infiltration and their association with clinical outcomes based on the tumor location. Repeating the analysis in the right and left-sided CRCs, showed that the CD8 expression in IM of right-sided CRCs was significantly associated with improved OS. No significant association between T cell infiltration and survival might imply that T cells fail to eliminate cancer cells in TME effectively. There are a wide variety of immunosuppressive mechanisms in the TME that can result in modulation and functional impairment of T cells. In the TME, infiltrated immune cells display a broad spectrum of dysfunctional states that are shaped by suppressive signals within the TME (43, 44). The role of these immune cells in tumor growth and progression is diverse and is tightly linked to the molecules and ligands they express (41). Among them, up-regulation of inhibitory ICPs is a hallmark of the tumor ecosystem resulting in immune cell dysfunction (9).

PD-1 is primarily introduced as an inhibitory receptor for the immune system, and its inhibition has been reported to restore immune activities in several malignancies (15). Nevertheless, some studies showed that PD-1 expression had been linked to favorable prognosis in several malignancies, including gastric, breast, small cell lung cancer, HPV-associated head and neck cancer, and CRC, as well (37, 45–49). Accordingly, the effect of this receptor on T cell function and tumor prognosis remains unclear. The results of our study showed that higher PD-1 expression in the IM was associated with lower T-stage (T1/2) in the total cohort and left-sided CRCs, lower M-stage (M0) in the total cohort and right-sided CRCs, lower TNM stage (I/II) just in the total cohort, lack of metastasis in the total cohort and both right and left-sided CRCs, lack of recurrence just in left-sided CRCs, and conversely with larger tumor size (≥5 cm) just in right-sided CRCs. Consistently, Saleh et al. showed that *PD-1* gene expression in both tumor tissue and blood circulation of patients with CRC is higher in the early stages of CRC (50). Higher expression of PD-1 in the early stages of the CRC, could implies that targeting PD-1 in these stages might be more effective.

The prognostic association of PD-1 expression in CRC has been also examined in several studies, however the results are inconsistent (17, 18, 23). Controversial results could be depending on some parameters such as tumor stage, location, or mismatch repair (MMR) status. Zengin et al. showed that high PD-1 expression could be an independent poor prognostic factor for recurrence-free survival (RFS) and OS in CRC patients in stage III (23). Contradictory, Li et al. showed that high PD-1 expression in TILs is an independent prognostic factor for improved OS and DFS in CRC patients, particularly for MMR-proficient subgroup (49). Lee et al. showed that the high PD-1 expression in TILs in MMR-deficient tumors is relined to enhanced RFS only when PD-L1 expression in tumor cells is low (21). Furthermore, Ahtiainen et al. found that having a high density of PD-1+ TILs was linked to better OS and DFS in CRC, regardless of the MMR status or immune cell score (17). Berntsson et al. showed that high PD-1 expression on immune cells is associated with improved OS, and this association depends on tumor location (18). These observations call into question the prognostic value of PD-1 in CRC. Similarly, our results showed that high PD-1 expression in the IM was associated with better OS; however, it was not statistically significant. Nevertheless, we observed that classifying patients based on their CD8 expression and tumor location, high PD-1 expression in IM was associated with improved OS only in the CD8 high expression subgroup.

Due to similar effects on survival, we next incorporated CD8 and PD-1 expressions patterns into a single score to investigate whether they might work together to improve prognostic power. As a result, prognostic models demonstrated that patients with high expressions of PD-1 and CD8 in the IM had better OS than other patients; however, it was not significant. This result indicated that PD-1+ CD8+ TILs had proper effector function in the IM of CRC.

The association between high PD-1 expression (with or without CD8) and better OS and lower stage of the disease is in contrast with the commonly defined role for this molecule as PD-1 generally is introduced as an inhibitory receptor and is a hallmark of T cell exhaustion. Due to the low PD-L1 expression in CRC tumors, it can be concluded that in the absence of PD-1 ligation to its ligand, the high expression of PD-1 in the TILs might reflect the effector phenotypes of these cells (21, 51). It has recently been demonstrated that PD-1 differentially affects cell proliferation, maturation, and transcript signatures among diverse immune cell populations. While naive T cells are inhibited following PD-1 ligation, T cells with effector and central memory phenotypes proliferate after ligation of PD-1 by its ligand (52). There is also evidence that PD-1 signaling is not essential for CD8+ T cell exhaustion (53). Moreover, recent studies have demonstrated that CD8+ T cells remain functional despite PD-1 expression in different tumors such as breast cancer, non-small cell lung cancer, and gastric cancer (54–56).

Tim3 is another inhibitory receptor that has been reported as a negative prognostic factor for tumors such as CRC, alone or in combination with PD-1 (20, 35). Little is known about the prognostic role of Tim3 in cancer. While Tim3 was associated with worse prognosis in non-small cell lung cancer and gastric cancer, it corelated with a better prognosis in breast cancer (57). To the best of our knowledge, this is the first study that evaluated the Tim3 expression on the immune cells in the IM and CT, according to the primary site of the tumor in CRC. our results indicated that, Tim3 expression was just observed on immune cells (innate and adaptive), while other studies on CRC reported its expression on tumor cells, as well (20, 29). We also observed that Tim3 expression is upregulated in tumor tissue comparing to normal-like adjacent tissue, and this expression in the CT was associated with higher M-stage (M1) in left-sided CRCs, older age in the total cohort, and left-sided CRCs. In addition, high expression of Tim3 in CT was associated with poor OS in the total cohort and left-sided CRCs (especially in patients with high infiltration of CD8+ TILs). In the adjusted model, Tim3 in CT remained an independent poor prognostic factor besides T-stage, M-stage, lymph node involvement, and tumor budding. These results are in concordance with previous studies showing that Tim3 expression is upregulated in tumor tissues and is associated with poor prognosis in patients with CRC, gastric cancer, prostate cancer, clear cell renal cancer, urothelial bladder cancer, and cervical cancer (29–34).

Regarding Tim3 expression on immune cells, previous studies on gastric cancer showed that Tim3 expressions on T cells (CD8+ and Treg) and NK cells, respectively, were associated with poor prognosis and advanced stages of the tumor (58, 59). Moreover, increased Tim3 expression on tumor-specific CD8+ T cells was associated with impaired CD8+ T cell function and poor prognosis in HBV-associated hepatocellular carcinoma (60) and prostate cancer (61). In CRC, it is reported that Tim3+CD8+ T cells are more prone to apoptosis than Tim3- CD8+ T cells (62). In addition, upregulation of Tim3 and PD-1 on CD8+ and CD4+ T cells is associated with the dysfunctionality of these cells and less IFNγ production (39). All of these studies indicate that overexpression of Tim3 in TME has inhibitory effects on immune responses against tumors. Paradoxically, Al-Badran et al. showed that Tim3 expression on stromal immune cells is associated with a better CRC prognosis (37). Similarly, we observed that the Tim3 expression on immune cells at the IM was higher in patients who had no metastasis and were alive.

To investigate the effects of PD-1 and Tim3 expression in different regions (CT and IM) on survival, we combined their expression levels in the IM and CT. This combination for Tim3 showed that patients with high expression of Tim3 in the CT while low expression in the IM, had significantly a poorer OS than other groups. These data indicated that the distribution of Tim3+ immune cells in the tumor could probably reflect different functions or differentially affect clinical outcomes and prognosis. The difference might be explained by the difference in the expression of Tim3 ligands such as galactin-9 in different tumor areas. On the other hand, this combination for PD-1 showed that patients with high expression of PD-1 in IM but low expression in CT had a better OS than other groups; however, it was not significant. This issue should be a topic that warrants further investigation in a larger population.

To evaluate the synergistic effect of these two inhibitory ICPs on survival, we combined their expression in CT and IM and repeated the analysis. We observed that patients with high expression of both PD-1 and Tim3 in IM but low PD-1 and Tim3 expression in CT had improved OS; however, their relations weren’t statistically significant, may be due to the small sample size. In this regard, Al-Badran et al. similarly showed that the combination of high expression of Tim3, LAG-3, and PD-1 on the stromal immune cells is associated with better outcomes in CRC patients (37). Contradictory, Kuai et al. showed that co-expression of PD-1 and Tim3 is associated with a worse prognosis in CRC (20). Based on these results and new data (63–65), PD-1 and Tim3 probably have a dual function, and some key factors affect the activity of these molecules in the TME. Thus, understanding the role of these molecules can be improved by identifying these key factors in the TME.

In conclusion, our findings suggest that the clinicopathological and prognostic significance of immune system-related markers such as CD8, PD-1, and Tim3 depends on the primary tumor sides. Such studies could potentially be helpful in patient clinical management, since our results suggest that the tumor side may be a factor in therapeutic decisions, including immunotherapy based on inhibitory receptors. We also noted that PD-1 and Tim3 expressions in different regions of the resected tumor have different prognostic impacts, which could be explained by the different properties of TME in each region. This finding should be more evaluated in future studies to confirm and determine the reasons for these differences. Overall, we showed that Tim3 could act as a prognostic factor and therapeutic target in CRC. This marker is probably a more preferred target for immunotherapy than PD-1, especially in left-sided CRCs.

## Data availability statement

The data used and/or analyzed during the current study are available in the CRC-ICM data set (66) (https://data.mendeley.com/datasets/h3fhg9zr47).

## Ethics statement

The studies involving human participants were reviewed and approved by school of medicine, Isfahan university of medical sciences (IR.MUI.MED.REC.1398.611). Written informed consent for participation was not required for this study in accordance with the national legislation and the institutional requirements.

## Author contributions

ZM and ST carried out the experiments. ZM wrote the main manuscript text and prepared figures. MS and AD carried out pathology sections analysis and data collection. ZM done data collection and statistical analysis. ZH helped in statistical analysis. ZF helped shape the research, analysis and manuscript edition. MR conceived the original idea and supervised the project and manuscript final edition. All authors contributed to the article and approved the submitted version.
