# Supplementary material for: Tim3 and PD-1 as a therapeutic and prognostic targets in colorectal cancer: Relationship with sidedness, clinicopathological parameters, and survival
Source: Front Oncol. 2023 Mar 23;13:1069696. doi: 10.3389/fonc.2023.1069696 (PMC10076872; doi:10.3389/fonc.2023.1069696)
Supplement: Supplementary file 1 [file Table_1.docx]

**Supplementary Table 1.** The mean expression of PD-1 and Tim3 according to Clinicopathological Parameters

| **Parameters** | **PD-1-CT** | | **PD-1-IM** | | **Tim3-CT** | | **Tim3-IM** | |
| --- | --- | --- | --- | --- | --- | --- | --- | --- |
|  | **Mean ± SD** | **P** | **Mean ± SD** | **P** | **Mean ± SD** | **P** | **Mean ± SD** | **P** |
| **Sex** | | | | | | | | |
| Male | 9.0241±9.83396 | 0.833 | 9.4146±7.23836 | 0.991 | 26.5181±15.21581 | 0.024* | 33.4878±16.35580 | 0.118 |
| Female | 7.6792±6.25046 |  | 8.7358±6.56960 |  | 32.7358±15.94962 |  | 38.2075±18.00359 |  |
| **Age** | | | | | | | | |
| <63 | 9.0923±9.52583 | 0.133 | 8.9844±6.69634 | 0.906 | 28.1538±15.323828 | 0.579 | 33.8462±17.89224 | 0.33 |
| >=63 | 7.9577±7.71721 |  | 9.2958±7.24548 |  | 29.662±16.19431 |  | 36.7286±16.36178 |  |
| **Tumor side** | | | | | | | | |
| Right | 8.6964±10.19968 | 0.932 | 9.9636±6.26266 | 0.175 | 29.6607±16.22823 | 0.817 | 39.2143±17.06215 | 0.032* |
| Left | 8.4474±7.42858 |  | 8.7632±7.51508 |  | 29.0132±15.55677 |  | 32.7333±16.91180 |  |
| **Tumor size** | | | | | | | | |
| <5 | 7.3214±6.19122 | 0.189 | 7.6909±5.95624 | 0.027* | 28.6786±14.62763 | 0.771 | 33.8571±17.33775 | 0.398 |
| ≥5 | 9.3590±9.98567 |  | 10.2821±7.46054 |  | 29.4872±16.60445 |  | 36.4286±17.18432 |  |
| **Differentiation grade** | | | | | | | | |
| Low grade | 7.6053±5.99128 | 0.377 | 9.0667±6.87206 | 0.797 | 30.2632±14.85249 | 0.272 | 36.9737±15.57945 | 0.209 |
| Moderate to high grade | 9.6333±11.03917 |  | 9.2500±7.13935 |  | 27.2667±16.78262 |  | 33.2373±18.83352 |  |
| **T stage** | | | | | | | | |
| T1/T2 | 9.9574±10.95238 | 0.047* | 9.9574±6.76293 | 0.211 | 28.1915±16.79239 | 0.688 | 34.3617±18.66807 | 0.629 |
| T3/T4 | 7.7303±7.03040 |  | 8.7159±7.07261 |  | 29.3371±15.24493 |  | 35.8636±16.30893 |  |
| **Lymph node involvement** | | | | | | | | |
| Absent | 8.6556±9.20816 | 0.969 | 9.2697±7.12991 | 0.878 | 29.1222±14.60016 | 0.852 | 36.5667±17.27507 | 0.241 |
| Present | 8.1957±7.40456 |  | 8.9130±6.70928 |  | 28.5870±17.94012 |  | 32.8889±16.70223 |  |
| **M stage** | | | | | | | | |
| M0 | 8.7742±8.89033 | 0.181 | 9.4878±7.10041 | 0.044* | 28.5565±15.96047 | 0.362 | 35.4553±17.44558 | 0.804 |
| M1 | 5.6667±4.18511 |  | 5.6667±4.18511 |  | 32.9167±13.22160 |  | 34.1667±13.78954 |  |

**Supplementary Table 1.** Continued

| **Parameters** | **PD-1-CT** | | **PD-1-IM** | | **Tim3-CT** | | **Tim3-IM** | |
| --- | --- | --- | --- | --- | --- | --- | --- | --- |
|  | **Mean ± SD** | **P^a^** | **Mean ± SD** | **P^a^** | **Mean ± SD** | **P** | **Mean ± SD** | **P** |
| **TNM stage** | | | | | | | | |
| I/II | 9.0000±9.39880 | 0.322 | 9.6627±7.17257 | 0.184 | 28.9405±14.98863 | 0.999 | 36.3214±17.72763 | 0.395 |
| III/IV | 7.6923±7.18533 |  | 8.3269±6.60873 |  | 28.9423±17.04482 |  | 33.7255±16.08860 |  |
| **Lymphovascular invasion (LVI**) | | | | | | | | |
| Absent | 9.4304±9.94173 | 0.17 | 9.8354±7.51141 | 0.233 | 29.5570±14.93983 | 0.593 | 37.1519±16.97818 | 0.145 |
| Present | 7.2105±6.19256 |  | 8.1786±6.04861 |  | 28.0877±16.89387 |  | 32.7857±17.12597 |  |
| **Perineural invasion** | | | | | | | | |
| Absent | 8.7387±9.00173 | 0.297 | 9.2818±7.30732 | 0.77 | 29.8288±16.51108 | 0.167 | 35.5545±17.50417 | 0.762 |
| Present | 7.4400±6.68381 |  | 8.5600±5.29213 |  | 25.0±11.18034 |  | 34.40±15.56706 |  |
| **Metastasis** | | | | | | | | |
| Absent | 9.3933±9.70194 | 0.035* | 10.3523±7.42788 | <0.001* | 28.8876±16.79789 | 0.701 | 37.9888±17.42223 | 0.038* |
| Present | 6.1176±4.7211 |  | 6.2353±5.21105 |  | 27.6471±13.66456 |  | 30.6061±16.94465 |  |
| **Recurrence** | | | | | | | | |
| Absent | 8.8557±9.48243 | 0.717 | 9.8854±7.45353 | 0.043* | 28.7216±16.01025 | 0.622 | 37.0206±17.47318 | 0.137 |
| Present | 7.9310±6.28451 |  | 7.3793±5.54471 |  | 27.069±15.09004 |  | 31.4286±17.15167 |  |
| **Survival** | | | | | | | | |
| Alive | 8.5696±9.56447 | 0.937 | 9.5769±6.69662 | 0.274 | 28.3671±16.76778 | 0.618 | 37.5128±17.89707 | 0.084 |
| Dead | 8.4035±7.17600 |  | 8.5614±7.33879 |  | 29.7368±14.31224 |  | 32.3684±15.64407 |  |
| **Tumor budding** | | | | | | | | |
| Low | 8.7416±9.19304 | 0.64 | 9.1250±6.98243 | 0.752 | 27.2022±13.89668 | 0.105 | 34.5000±16.39456 | 0.437 |
| High | 8.0426±7.47156 |  | 9.1915±7.01129 |  | 32.2340±18.46879 |  | 36.9149±18.46128 |  |
| **Tertiary lymphoid structure (TLS)** | | | | | | | | |
| Absent | 7.4135±6.09410 | 0.184 | 8.0874±6.03276 | 0.002* | 29.0481±16.27935 | 0.887 | 33.6505±17.54362 | 0.039* |
| Present | 12.0313±13.52771 |  | 12.5625±8.61708 |  | 28.5938±14.09841 |  | 40.7813±14.59725 |  |

CT: Center of the tumor

IM: Invasive margin of the tumor

*: Statistically significant

^a^: A T-test performed with normalized data (Ln or Sqrt) to obtain the P value.
