# Supplementary material for: Tim3 and PD-1 as a therapeutic and prognostic targets in colorectal cancer: Relationship with sidedness, clinicopathological parameters, and survival
Source: Front Oncol. 2023 Mar 23;13:1069696. doi: 10.3389/fonc.2023.1069696 (PMC10076872; doi:10.3389/fonc.2023.1069696)
Supplement: Supplementary file 2 [file Table_2.docx]

**Supplementary Table 2.** Associations between PD-1 expression in TILs and clinicopathological parameters stratified by primary tumor side

| **Parameters** | **Total cohort** | | | | | | **Right-side** | | | | | | **Left-side** | | | | | |
| --- | --- | --- | --- | --- | --- | --- | --- | --- | --- | --- | --- | --- | --- | --- | --- | --- | --- | --- |
|  | **PD-1-CT** | | **P** | **PD-1-IM** | | **P** | **PD-1-CT** | | **P** | **PD-1-IM** | | **P** | **PD-1-CT** | | **P** | **PD-1-IM** | | **P** |
| n (%) | **Low** | **High** |  | **Low** | **High** |  | **Low** | **High** |  | **Low** | **High** |  | **Low** | **High** |  | **Low** | **High** |  |
| **Sex** | | | | | | | | | | | | | | | | | | |
| Male | 49(59) | 34(64.2) | 0.551 | 35(55.6) | 47(65.3) | 0.248 | 21(58.3) | 12(60) | 0.903 | 10(50) | 22(62.9) | 0.352 | 26(57.8) | 21(67.7) | 0.38 | 23(57.5) | 24(66.7) | 0.411 |
| Female | 34(41) | 19(35.8) |  | 28(44.4) | 25(34.7) |  | 15(41.7) | 8(40) |  | 10(50) | 13(37.1) |  | 19(42.2) | 10(32.3) |  | 17(42.5) | 12(33.3) |  |
| **Age** | | | | | | | | | | | | | | | | | | |
| <63 | 38(45.8) | 27(50.9) | 0.557 | 33(52.4) | 31(43.1) | 0.279 | 16(44.4) | 11(55) | 0.449 | 11(55) | 15(42.9) | 0.386 | 22(48.9) | 16(51.6) | 0.815 | 22(55) | 16(44.4) | 0.358 |
| ≥63 | 45(54.2) | 26(49.1) |  | 30(47.6) | 41(56.9) |  | 20(55.6) | 9(45) |  | 9(45) | 20(57.1) |  | 23(51.1) | 15(48.4) |  | 18(45) | 20(55.6) |  |
| **Tumor side** | | | | | | | | | | | | | | | | | | |
| Right | 36(44.4) | 20(39.2) | 0.554 | 20(33.3) | 35(49.3) | 0.065 |  | | | | | | | | | | | |
| Left | 45(55.6) | 31(60.8) |  | 40(66.7) | 36(50.7) |  |  | | | | | | | | | | | |
| Unknown | 2 | 2 |  | 3 | 1 |  |  | | | | | | | | | | | |
| **Tumor size** | | | | | | | | | | | | | | | | | | |
| <5 | 38(46.3) | 18(34.6) | 0.18 | 30(48.4) | 25(35.2) | 0.124 | 13(37.1) | 6(30) | 0.592 | 10(52.6) | 8(22.9) | 0.027* | 24(53.3) | 11(35.5) | 0.125 | 18(45) | 17(47.2) | 0.846 |
| ≥5 | 44(53.7) | 34(65.4) |  | 32(51.6) | 46(64.8) |  | 22(62.9) | 14(70) |  | 9(47.4) | 27(77.1) |  | 21(46.7) | 20(64.5) |  | 22(55) | 19(52.8) |  |
| Unknown | 1 | 1 |  | 1 | 1 |  | 1 |  |  | 1 |  |  |  |  |  |  |  |  |
| **Differentiation grade** | | | | | | | | | | | | | | | | | | |
| Low grade | 47(56.6) | 29(54.7) | 0.827 | 35(55.6) | 40(55.6) | 1 | 21(58.3) | 10(50) | 0.548 | 11(55) | 19(54.3) | 0.959 | 25(55.6) | 18(58.1) | 0.828 | 23(57.5) | 20(55.6) | 0.864 |
| moderate to high grade | 36(43.4) | 24(45.3) |  | 28(44.4) | 32(44.4) |  | 15(41.7) | 10(50) |  | 9(45) | 16(45.7) |  | 20(44.4) | 13(41.9) |  | 17(42.5) | 16(44.4) |  |
| **T stage** | | | | | | | | | | | | | | | | | | |
| T1/T2 | 25(30.1) | 22(41.5) | 0.173 | 16(25.4) | 31(43.1) | 0.032* | 12(33.3) | 8(40) | 0.618 | 6(30) | 14(40) | 0.458 | 12(26.7) | 14(45.2) | 0.095 | 9(22.5) | 17(47.2) | 0.023* |
| T3/T4 | 58(69.9) | 31(58.5) |  | 47(74.6) | 41(56.9) |  | 24(66.7) | 12(60) |  | 14(70) | 21(60) |  | 33(73.3) | 17(54.8) |  | 31(77.5) | 19(52.8) |  |
| **Lymph node involvement** | | | | | | | | | | | | | | | | | | |
| Absent | 56(67.5) | 34(64.2) | 0.69 | 38(60.3) | 51(70.8) | 0.198 | 28(77.8) | 11(55) | 0.076 | 13(65) | 25(71.4) | 0.62 | 27(60) | 21(67.7) | 0.492 | 23(57.5) | 25(69.4) | 0.281 |
| Present | 27(32.5) | 19(35.8) |  | 25(39.7) | 21(29.2) |  | 8(22.2) | 9(45) |  | 7(35) | 10(28.6) |  | 18(40) | 10(32.3) |  | 17(42.5) | 11(30.6) |  |
| **M stage** | | | | | | | | | | | | | | | | | | |
| M0 | 74(89.2) | 50(94.3) | 0.299 | 54(85.7) | 69(95.8) | 0.039* | 32(88.9) | 20(100) | 0.285 | 16(80) | 35(100) | 0.014* | 40(88.9) | 28(90.3) | 1 | 35(87.5) | 33(91.7) | 0.715 |
| M1 | 9(10.8) | 3(5.7) |  | 9(14.3) | 3(4.2) |  | 4(11.1) | 0(0) |  | 4(20) | 0(0) |  | 5(11.1) | 3(9.7) |  | 5(12.5) | 3(8.3) |  |

**Supplementary Table 2.** Continue

| **Parameters** | **Total cohort** | | | | | | **Right-side** | | | | | | **Left-side** | | | | | |
| --- | --- | --- | --- | --- | --- | --- | --- | --- | --- | --- | --- | --- | --- | --- | --- | --- | --- | --- |
|  | **PD-1-CT** | | **P** | **PD-1-IM** | | **P** | **PD-1-CT** | | **P** | **PD-1-IM** | | **P** | **PD-1-CT** | | **P** | **PD-1-IM** | | **P** |
| N (%) | **Low** | **High** |  | **Low** | **High** |  | **Low** | **High** |  | **Low** | **High** |  | **Low** | **High** |  | **Low** | **High** |  |
| **TNM stage** | | | | | | | | | | | | | | | | | | |
| I/II | 51(61.4) | 33(62.3) | 0.924 | 33(52.4) | 50(69.4) | 0.042* | 25(69.4) | 11(55) | 0.28 | 10(50) | 25(71.4) | 0.112 | 25(55.6) | 20(64.5) | 0.435 | 21(52.5) | 24(66.7) | 0.21 |
| III/IV | 32(38.6) | 20(37.7) |  | 30(47.6) | 22(30.6) |  | 11(30.6) | 9(45) |  | 10(50) | 10(28.6) |  | 20(44.4) | 11(35.5 |  | 19(47.5) | 12(33.3) |  |
| **Lymphovascular invasion (LVI**) | | | | | | | | | | | | | | | | | | |
| Absent | 47(56.6) | 32(60.4) | 0.665 | 33(52.4) | 46(63.9) | 0.176 | 23(63.9) | 13(65) | 0.934 | 12(60) | 24(68.6) | 0.52 | 24(53.3) | 19(61.3) | 0.492 | 21(52.5) | 22(61.1) | 0.45 |
| Present | 36(43.4) | 21(39.6) |  | 30(47.6) | 26(36.1) |  | 13(36.1) | 7(35) |  | 8(40) | 11(31.4) |  | 21(46.7) | 12(38.7) |  | 19(47.5) | 14(38.9) |  |
| **Perineural invasion** | | | | | | | | | | | | | | | | | | |
| Absent | 67(80.7) | 44(83) | 0.763 | 52(82.5) | 58(80.6) | 0.767 | 30(83.3) | 18(90) | 0.697 | 16(80) | 31(88.6) | 0.443 | 35(77.8) | 25(80.6) | 0.763 | 33(82.5) | 27(75) | 0.423 |
| Present | 16(19.3) | 9(17) |  | 11(17.5) | 14(19.4) |  | 6(16.7) | 2(10) |  | 4(20) | 4(11.4) |  | 10(22.2) | 6(19.4) |  | 7(17.5) | 9(25) |  |
| **Metastasis** | | | | | | | | | | | | | | | | | | |
| Absent | 50(66.7) | 39(81.3) | 0.078 | 33(57.9) | 55(84.6) | 0.001* | 24(72.7) | 16(80) | 0.744 | 11(57.9) | 28(84.8) | 0.047* | 24(60) | 21(80.8) | 0.077 | 19(54.3) | 26(83.9) | 0.01* |
| Present | 25(33.3) | 9(18.8) |  | 24(42.1) | 10(15.4) |  | 9(27.3) | 4(20) |  | 8(42.1) | 5(15.2) |  | 16(40) | 5(19.2) |  | 16(45.7) | 5(16.1) |  |
| Unknown | 8 | 5 |  | 6 | 7 |  |  |  |  | 1 | 2 |  |  |  |  | 5 | 5 |  |
| **Recurrence** | | | | | | | | | | | | | | | | | | |
| Absent | 58(76.3) | 39(78) | 0.826 | 41(70.7) | 55(82.1) | 0.132 | 28(84.8) | 14(70) | 0.296 | 14(77.8) | 27(79.4) | 1 | 28(68.3) | 24(85.7) | 0.099 | 24(64.9) | 28(87.5) | 0.03* |
| Present | 18(23.7) | 11(22) |  | 17(29.3) | 12(17.9) |  | 5(15.2) | 6(30) |  | 4(22.2) | 7(20.6) |  | 13(31.7) | 4(14.3) |  | 13(35.1) | 4(12.5) |  |
| Unknown | 7 | 3 |  | 5 | 5 |  | 3 |  |  | 2 | 1 |  | 4 | 3 |  | 3 | 4 |  |
| **Survival** | | | | | | | | | | | | | | | | | | |
| Alive | 49(59) | 30(56.6) | 0.779 | 33(52.4) | 45(62.5) | 0.235 | 24(66.7) | 14(70) | 0.798 | 12(60) | 25(71.4) | 0.385 | 24(53.3) | 16(51.6) | 0.883 | 20(50) | 20(55.6) | 0.628 |
| Dead | 34(41) | 23(43.4) |  | 30(47.6) | 27(37.5) |  | 12(33.3) | 6(30) |  | 8(40) | 10(28.6) |  | 21(46.7) | 15(48.4) |  | 20(50) | 16(44.4) |  |
| **Tumor budding** | | | | | | | | | | | | | | | | | | |
| Low | 53(63.9) | 36(67.9) | 0.627 | 40(63.5) | 48(66.7) | 0.699 | 22(61.1) | 15(75) | 0.293 | 12(60) | 24(68.6) | 0.52 | 29(64.4) | 19(61.3) | 0.779 | 25(62.5) | 23(63.9) | 0.9 |
| High | 30(36.1) | 17(32.1) |  | 23(36.5) | 24(33.3) |  | 14(38.9) | 5(25) |  | 8(40) | 11(31.4) |  | 16(35.6) | 12(38.7) |  | 15(37.5) | 13(36.1) |  |
| **Tertiary lymphoid structure (TLS)** | | | | | | | | | | | | | | | | | | |
| Absent | 67(80.7) | 37(69.8) | 0.143 | 53(84.1) | 50(69.4) | 0.045* | 30(83.3) | 12(60) | 0.053 | 17(85) | 24(68.6) | 0.178 | 35(77.8) | 24(77.4) | 0.971 | 33(82.5) | 26(72.2) | 0.283 |
| Present | 16(19.3) | 16(30.2) |  | 10(15.9) | 22(30.6) |  | 6(16.7) | 8(40) |  | 3(15) | 11(31.4) |  | 10(22.2) | 7(22.6) |  | 7(17.5) | 10(27.8) |  |

**Supplementary Table 2.** Continued

| **Parameters** | **Total cohort** | | | | | | **Right-side** | | | | | | **Left-side** | | | | | |
| --- | --- | --- | --- | --- | --- | --- | --- | --- | --- | --- | --- | --- | --- | --- | --- | --- | --- | --- |
|  | **PD-1-CT** | | **P** | **PD-1-IM** | | **P** | **PD-1-CT** | | **P** | **PD-1-IM** | | **P** | **PD-1-CT** | | **P** | **PD-1-IM** | | **P** |
| n (%) | **Low** | **High** |  | **Low** | **High** |  | **Low** | **High** |  | **Low** | **High** |  | **Low** | **High** |  | **Low** | **High** |  |
| **CD8.CT** | | | | | | | | | | | | | | | | | | |
| Low | 49(59.8) | 20(37.7) | 0.012* |  |  |  | 23(63.9) | 8(40.0) | 0.085 |  |  |  | 25(56.8) | 10(32.3) | 0.036* |  |  |  |
| High | 33(40.2) | 33(62.3) |  |  |  |  | 13(36.1) | 12(60.0) |  |  |  |  | 19(43.2) | 21(67.7) |  |  |  |  |
| **CD3.CT** | | | | | | | | | | | | | | | | | | |
| Low | 56(67.5) | 25(47.2) | 0.019* |  |  |  | 26(72.2) | 9(45.0) | 0.044* |  |  |  | 28(62.2) | 15(48.4) | 0.232 |  |  |  |
| High | 27(32.5) | 28(52.8) |  |  |  |  | 10(27.8) | 11(55.0) |  |  |  |  | 17(37.8) | 16(51.6) |  |  |  |  |
| **CD8.IM** | | | | | | | | | | | | | | | | | | |
| Low |  |  |  | 48(76.2) | 37(51.4) | 0.003* |  |  |  | 17(85.0) | 19(54.3) | 0.021* |  |  |  | 29(72.5) | 17(47.2) | 0.024* |
| High |  |  |  | 15(23.8) | 35(48.6) |  |  |  |  | 3(15.0) | 16(45.7) |  |  |  |  | 11(27.5) | 19(52.8) |  |
| **CD3.IM** | | | | | | | | | | | | | | | | | | |
| Low |  |  |  | 41(65.1) | 33(45.8) | 0.025* |  |  |  | 14(70.0) | 14(40.0) | 0.032* |  |  |  | 26(65.0) | 19(52.8) | 0.279 |
| High |  |  |  | 22(34.9) | 39(54.2) |  |  |  |  | 6(30.0) | 21(60.0) |  |  |  |  | 14(35.0) | 17(47.2) |  |

CT: Center of the tumor

IM: Invasive margin of the tumor

*: Statistically significant
