# Supplementary material for: Tim3 and PD-1 as a therapeutic and prognostic targets in colorectal cancer: Relationship with sidedness, clinicopathological parameters, and survival
Source: Front Oncol. 2023 Mar 23;13:1069696. doi: 10.3389/fonc.2023.1069696 (PMC10076872; doi:10.3389/fonc.2023.1069696)
Supplement: Supplementary file 3 [file Table_3.docx]

**Supplementary Table 3.** Associations between Tim3 expression in TIICs and clinicopathological parameters stratified by primary tumor side

| **Parameters** | **Total cohort** | | | | | | **Right-side** | | | | | | **Left-side** | | | | | |
| --- | --- | --- | --- | --- | --- | --- | --- | --- | --- | --- | --- | --- | --- | --- | --- | --- | --- | --- |
|  | **Tim3-CT** | | **P** | **Tim3-IM** | | **P** | **Tim3-CT** | | **P** | **Tim3-IM** | | **P** | **Tim3-CT** | | **P** | **Tim3-IM** | | **P** |
| N (%) | **Low** | **High** |  | **Low** | **High** |  | **Low** | **High** |  | **Low** | **High** |  | **Low** | **High** |  | **Low** | **High** |  |
| **Sex** | | | | | | | | | | | | | | | | | | |
| Male | 49(62.8) | 34(58.6) | 0.619 | 28(75.7) | 54(55.1) | 0.029* | 20(62.5) | 13(54.2) | 0.53 | 8(72.7) | 25(55.6) | 0.496 | 26(61.9) | 21(61.8) | 0.99 | 19(76) | 27(54) | 0.065 |
| Female | 29(37.2) | 24(41.4) |  | 9(24.3) | 44(44.9) |  | 12(37.5) | 11(45.8) |  | 3(27.3) | 20(44.4) |  | 16(38.1) | 13(38.2) |  | 6(24) | 23(46) |  |
| **Age** | | | | | | | | | | | | | | | | | | |
| <63 | 43(55.1) | 22(37.9) | 0.047* | 19(51.4) | 46(46.9) | 0.647 | 16(50) | 11(45.8) | 0.757 | 7(63.6) | 20(44.4) | 0.253 | 27(64.3) | 11(32.4) | 0.006* | 12(48) | 26(52) | 0.744 |
| ≥63 | 35(44.9) | 36(62.1) |  | 18(48.6) | 52(53.1) |  | 16(50) | 13(54.2) |  | 4(36.4) | 25(55.6) |  | 15(35.7) | 23(67.6) |  | 13(52) | 24(48) |  |
| **Tumor side** | | | | | | | | | | | | | | | | | | |
| Right | 32(43.2) | 24(41.4) | 0.83 | 11(30.6) | 45(47.4) | 0.082 |  | | | | | | | | | | | |
| Left | 42(58.8) | 34(58.6) |  | 25(69.4) | 50(52.6) |  |  |  |  |  |  |  |  |  |  |  |  |  |
| Unknown | 4 |  |  | 1 | 3 |  |  |  |  |  |  |  |  |  |  |  |  |  |
| **Tumor size** | | | | | | | | | | | | | | | | | | |
| <5 | 30(39.5) | 26(44.8) | 0.534 | 14(37.8) | 42(43.8) | 0.536 | 10(32.3) | 9(37.5) | 0.685 | 5(45.5) | 14(31.8) | 0.485 | 18(42.9) | 17(50) | 0.534 | 8(32) | 27(54) | 0.072 |
| ≥5 | 46(60.5) | 32(55.2) |  | 23(62.2) | 54(56.3) |  | 21(67.7) | 15(62.5) |  | 6(54.5) | 30(68.2) |  | 24(57.1) | 17(50) |  | 17(68) | 23(46) |  |
| Unknown | 2 |  |  |  | 2 |  | 1 |  |  |  | 1 |  |  |  |  |  |  |  |
| **Differentiation grade** | | | | | | | | | | | | | | | | | | |
| Low grade | 42(53.8) | 34(58.6) | 0.579 | 19(51.4) | 57(58.2) | 0.477 | 17(53.1) | 14(58.3) | 0.698 | 5(45.5) | 26(57.8) | 0.514 | 23(54.8) | 20(58.8) | 0.722 | 14(56) | 29(58) | 0.869 |
| Moderate to high grade | 36(46.2) | 24(41.4) |  | 18(48.6) | 41(41.8) |  | 15(46.9) | 10(41.7) |  | 6(54.5) | 19(42.2) |  | 19(45.2) | 14(41.2) |  | 11(44) | 21(42) |  |
| **T stage** | | | | | | | | | | | | | | | | | | |
| T1/T2 | 27(34.6) | 20(34.5) | 0.987 | 16(43.2) | 31(31.6) | 0.207 | 11(34.4) | 9(37.5) | 0.809 | 3(27.3) | 17(37.8) | 0.728 | 15(35.7) | 11(32.4) | 0.759 | 12(48) | 14(28) | 0.086 |
| T3/T4 | 51(65.4) | 38(65.5) |  | 21(56.8) | 67(68.4) |  | 21(65.6) | 15(62.5) |  | 8(72.7) | 28(62.2) |  | 27(64.3) | 23(67.6) |  | 13(52) | 36(72) |  |
| **Lymph node involvement** | | | | | | | | | | | | | | | | | | |
| Absent | 52(66.7) | 38(65.5) | 0.889 | 23(62.2) | 67(68.4) | 0.495 | 23(71.9) | 16(66.7) | 0.675 | 7(63.6) | 32(71.1) | 0.719 | 26(61.9) | 22(64.7) | 0.801 | 15(60) | 33(66) | 0.61 |
| Present | 26(33.3) | 20(34.5) |  | 14(37.8) | 31(31.6) |  | 9(28.1) | 8(33.3) |  | 4(36.4) | 13(28.9) |  | 16(38.1) | 12(35.3) |  | 10(40) | 17(34) |  |
| **M stage** | | | | | | | | | | | | | | | | | | |
| M0 | 74(94.9) | 50(86.2) | 0.078 | 35(94.6) | 88(89.8) | 0.511 | 29(90.6) | 23(95.8) | 0.627 | 10(90.9) | 42(93.3) | 1 | 41(97.6) | 27(79.4) | 0.019* | 24(96) | 43(86) | 0.256 |
| M1 | 4(5.1) | 8(13.8) |  | 2(5.4) | 10(10.2) |  | 3(9.4) | 1(4.2) |  | 1(9.1) | 3(6.7) |  | 1(2.4) | 7(20.6) |  | 1(4) | 7(14) |  |

**Supplementary Table 3.** Continued

| **Parameters** | **Total cohort** | | | | | | **Right-side** | | | | | | **Left-side** | | | | | |
| --- | --- | --- | --- | --- | --- | --- | --- | --- | --- | --- | --- | --- | --- | --- | --- | --- | --- | --- |
|  | **Tim3-CT** | | **P** | **Tim3-IM** | | **P** | **Tim3-CT** | | **P** | **Tim3-IM** | | **P** | **Tim3-CT** | | **P** | **Tim3-IM** | | **P** |
| N (%) | **Low** | **High** |  | **Low** | **High** |  | **Low** | **High** |  | **Low** | **High** |  | **Low** | **High** |  | **Low** | **High** |  |
| **TNM stage** | | | | | | | | | | | | | | | | | | |
| I/II | 50(64.1) | 34(58.6) | 0.515 | 23(62.2) | 61(62.2) | 0.993 | 21(65.6) | 15(62.5) | 0.809 | 7(63.6) | 29(64.4) | 1 | 26(61.9) | 19(55.9) | 0.595 | 15(60) | 30(60) | 1 |
| III/IV | 28(35.9) | 24(41.4) |  | 14(37.8) | 37(37.8) |  | 11(34.4) | 9(37.5) |  | 4(36.4) | 16(35.6) |  | 16(38.1) | 16(44.1) |  | 10(40) | 20(40) |  |
| **Lymphovascular invasion (LVI**) | | | | | | | | | | | | | | | | | | |
| Absent | 42(53.8) | 37(63.8) | 0.245 | 17(45.9) | 62(63.3) | 0.068 | 20(62.5) | 16(66.7) | 0.747 | 5(45.5) | 31(68.9) | 0.174 | 22(52.4) | 21(61.8) | 0.412 | 12(48) | 31(62) | 0.248 |
| Present | 36(46.2) | 21(36.2) |  | 20(54.1) | 36(36.7) |  | 12(37.5) | 8(33.3) |  | 6(54.5) | 14(31.1) |  | 20(47.6) | 13(38.2) |  | 13(52) | 19(38) |  |
| **Perineural invasion** | | | | | | | | | | | | | | | | | | |
| Absent | 60(76.9) | 51(87.9) | 0.101 | 31(83.8) | 79(80.6) | 0.672 | 27(84.4) | 21(87.5) | 1 | 11(100) | 37(82.2) | 0.333 | 30(71.4) | 30(88.2) | 0.074 | 19(76) | 40(80) | 0.69 |
| Present | 18(23.1) | 7(12.1) |  | 6(16.2) | 19(19.4) |  | 5(15.6) | 3(12.5) |  | 0(0) | 8(17.8) |  | 12(28.6) | 4(11.8) |  | 6(24) | 10(20) |  |
| **Metastasis** | | | | | | | | | | | | | | | | | | |
| Absent | 57(77) | 32(65.3) | 0.155 | 20(62.5) | 69(76.7) | 0.121 | 23(76.7) | 17(73.9) | 0.817 | 5(55.6) | 35(79.5) | 0.199 | 30(75) | 15(57.7) | 0.14 | 14(63.6) | 31(72.1) | 0.485 |
| Present | 17(23) | 17(34.7) |  | 12(37.5) | 21(23.3) |  | 7(23.3) | 6(26.1) |  | 4(44.4) | 9(20.5) |  | 10(25) | 11(42.3) |  | 8(36.4) | 12(27.9) |  |
| Unknown | 4 | 9 |  | 5 | 8 |  | 2 | 1 |  | 2 | 1 |  | 2 | 8 |  | 3 | 7 |  |
| **Recurrence** | | | | | | | | | | | | | | | | | | |
| Absent | 60(80) | 37(72.5) | 0.329 | 23(69.7) | 74(80.4) | 0.204 | 26(86.7) | 16(69.6) | 0.177 | 6(66.7) | 36(81.8) | 0.372 | 31(75.6) | 21(75) | 0.954 | 16(69.6) | 36(80) | 0.337 |
| Present | 15(20) | 14(27.5) |  | 10(30.3) | 18(19.6) |  | 4(13.3) | 7(30.4) |  | 3(33.3) | 8(18.2) |  | 10(24.4) | 7(25) |  | 7(30.4) | 9(20) |  |
| Unknown | 3 | 7 |  | 4 | 6 |  | 2 | 1 |  | 2 | 1 |  | 1 | 6 |  | 2 | 5 |  |
| **Survival** | | | | | | | | | | | | | | | | | | |
| Alive | 52(66.7) | 27(46.6) | 0.019* | 20(54.1) | 58(59.2) | 0.59 | 24(75) | 14(58.3) | 0.186 | 8(72.7) | 30(66.7) | 1 | 27(64.3) | 13(38.2) | 0.024* | 11(44) | 28(56) | 0.327 |
| Dead | 26(33.3) | 31(53.4) |  | 17(45.9) | 40(40.8) |  | 8(25) | 10(41.7) |  | 3(27.3) | 15(33.3) |  | 15(35.7) | 21(61.8) |  | 14(56) | 22(44) |  |
| **Tumor budding** | | | | | | | | | | | | | | | | | | |
| Low | 54(69.2) | 35(60.3) | 0.281 | 26(70.3) | 62(63.3) | 0.446 | 21(65.6) | 16(66.7) | 0.935 | 7(63.6) | 30(66.7) | 1 | 29(69) | 19(55.9) | 0.237 | 18(72) | 29(58) | 0.237 |
| High | 24(30.8) | 23(39.7) |  | 11(29.7) | 36(36.7) |  | 11(34.4) | 8(33.3) |  | 4(36.4) | 15(33.3) |  | 13(31) | 15(44.1) |  | 7(28) | 21(42) |  |
| **Tertiary lymphoid structure (TLS)** | | | | | | | | | | | | | | | | | | |
| Absent | 60(76.9) | 44(75.9) | 0.885 | 33(89.2) | 70(71.4) | 0.03* | 25(78.1) | 17(70.8) | 0.533 | 11(100) | 31(68.9) | 0.049* | 32(76.2) | 27(79.4) | 0.738 | 21(84) | 37(74) | 0.33 |
| Present | 18(23.1) | 14(24.1) |  | 4(10.8) | 28(28.6) |  | 7(21.9) | 7(29.2) |  | 0(0) | 14(31.1) |  | 10(23.8) | 7(20.6) |  | 4(16) | 13(26) |  |

**Supplementary Table 3.** Continued

| **Parameters** | **Total cohort** | | | | | | **Right-side** | | | | | | **Left-side** | | | | | |
| --- | --- | --- | --- | --- | --- | --- | --- | --- | --- | --- | --- | --- | --- | --- | --- | --- | --- | --- |
|  | **Tim3-CT** | | **P** | **Tim3-IM** | | **P** | **Tim3-CT** | | **P** | **Tim3-IM** | | **P** | **Tim3-CT** | | **P** | **Tim3-IM** | | **P** |
| n (%) | **Low** | **High** |  | **Low** | **High** |  | **Low** | **High** |  | **Low** | **High** |  | **Low** | **High** |  | **Low** | **High** |  |
| **PD-1.CT** | | | | | | | | | | | | | | | | | | |
| Low | 57(73.1) | 26(44.8) | 0.001* |  |  |  | 25(78.1) | 11(45.8) | 0.013* |  |  |  | 30(71.4) | 15(44.1) | 0.016* |  |  |  |
| High | 21(26.9) | 32(55.2) |  |  |  |  | 7(21.9) | 13(54.2) |  |  |  |  | 12(28.6) | 19(55.9) |  |  |  |  |
| **CD8.CT** | | | | | | | | | | | | | | | | | | |
| Low | 45(58.4) | 24(41.4) | 0.05 |  |  |  | 20(62.5) | 11(45.8) | 0.214 |  |  |  | 22(53.7) | 13(38.2) | 0.183 |  |  |  |
| High | 32(41.6) | 34(58.6) |  |  |  |  | 12(37.5) | 13(54.2) |  |  |  |  | 19(46.3) | 21(61.8) |  |  |  |  |
| **CD3.CT** | | | | | | | | | | | | | | | | | | |
| Low | 55(70.5) | 26(44.8) | 0.003* |  |  |  | 24(75.0) | 11(45.8) | 0.026* |  |  |  | 28(66.7) | 15(44.1) | 0.049* |  |  |  |
| High | 23(29.5) | 32(55.2) |  |  |  |  | 8(25.0) | 13(54.2%) |  |  |  |  | 14(33.3) | 19(55.9) |  |  |  |  |
| **PD-1.IM** | | | | | | | | | | | | | | | | | | |
| Low |  |  |  | 23(63.9) | 39(39.8) | 0.013* |  |  |  | 3(30.0) | 17(37.8) | 0.731 |  |  |  | 19(76.0) | 20(40.0) | 0.003* |
| High |  |  |  | 13(36.1) | 59(60.2) |  |  |  |  | 7(70.0) | 28(62.2) |  |  |  |  | 6(24.0) | 30(60.0) |  |
| **CD8.IM** | | | | | | | | | | | | | | | | | | |
| Low |  |  |  | 28(75.7) | 57(58.2) | 0.06 |  |  |  | 6(54.5) | 30(66.7) | 0.497 |  |  |  | 21(84.0) | 25(50.0) | 0.004* |
| High |  |  |  | 9(24.3) | 41(41.8) |  |  |  |  | 5(45.5) | 15(33.3) |  |  |  |  | 4(16.0) | 25(50.0) |  |
| **CD3.IM** | | | | | | | | | | | | | | | | | | |
| Low |  |  |  | 26(70.3) | 48(49.0) | 0.027* |  |  |  | 7(63.6) | 22(48.9) | 0.38 |  |  |  | 18(72.0) | 26(52.0) | 0.097 |
| High |  |  |  | 11(29.7) | 50(51.0) |  |  |  |  | 4(36.4) | 23(51.1) |  |  |  |  | 7(28.0) | 24(48.0) |  |

CT: Center of the tumor

IM: Invasive margin of the tumor

*: Statistically significant
